# Intra-operative applications of augmented reality in glioma surgery: a systematic review

**DOI:** 10.3389/fsurg.2023.1245851

**Published:** 2023-08-21

**Authors:** Anya Ragnhildstveit, Chao Li, Mackenzie H. Zimmerman, Michail Mamalakis, Victoria N. Curry, Willis Holle, Noor Baig, Ahmet K. Uğuralp, Layth Alkhani, Zeliha Oğuz-Uğuralp, Rafael Romero-Garcia, John Suckling

**Affiliations:** ^1^ Integrated Research Literacy Group, Draper, UT, United States; ^2^ Department of Psychiatry, University of Cambridge, Cambridge, England; ^3^ Department of Clinical Neurosciences, University of Cambridge, Cambridge, England; ^4^ Department of Applied Mathematics and Theoretical Physics, University of Cambridge, Cambridge, England; ^5^ Department of Bioengineering, University of Pennsylvania, Philadelphia, PA, United States; ^6^ Department of Physics and Astronomy, The University of Utah, Salt Lake City, UT, United States; ^7^ Department of Molecular and Cellular Biology, Harvard University, Cambridge, MA, United States; ^8^ Department of Biology, Stanford University, Stanford, CA, United States; ^9^ Instituto de Biomedicina de Sevilla (IBiS) HUVR/CSIC/Universidad de Sevilla/CIBERSAM, ISCIII, Dpto. de Fisiología Médica y Biofísica

**Keywords:** augmented reality, brain tumor, glioma, mixed reality, neuronavigation, neurosurgery, systematic review, virtual reality

## Abstract

**Background:**

Augmented reality (AR) is increasingly being explored in neurosurgical practice. By visualizing patient-specific, three-dimensional (3D) models in real time, surgeons can improve their spatial understanding of complex anatomy and pathology, thereby optimizing intra-operative navigation, localization, and resection. Here, we aimed to capture applications of AR in glioma surgery, their current status and future potential.

**Methods:**

A systematic review of the literature was conducted. This adhered to the Preferred Reporting Items for Systematic Reviews and Meta-Analyses (PRISMA) guideline. PubMed, Embase, and Scopus electronic databases were queried from inception to October 10, 2022. Leveraging the Population, Intervention, Comparison, Outcomes, and Study design (PICOS) framework, study eligibility was evaluated in the qualitative synthesis. Data regarding AR workflow, surgical application, and associated outcomes were then extracted. The quality of evidence was additionally examined, using hierarchical classes of evidence in neurosurgery.

**Results:**

The search returned 77 articles. Forty were subject to title and abstract screening, while 25 proceeded to full text screening. Of these, 22 articles met eligibility criteria and were included in the final review. During abstraction, studies were classified as “development” or “intervention” based on primary aims. Overall, AR was qualitatively advantageous, due to enhanced visualization of gliomas and critical structures, frequently aiding in maximal safe resection. Non-rigid applications were also useful in disclosing and compensating for intra-operative brain shift. Irrespective, there was high variance in registration methods and measurements, which considerably impacted projection accuracy. Most studies were of low-level evidence, yielding heterogeneous results.

**Conclusions:**

AR has increasing potential for glioma surgery, with capacity to positively influence the onco-functional balance. However, technical and design limitations are readily apparent. The field must consider the importance of consistency and replicability, as well as the level of evidence, to effectively converge on standard approaches that maximize patient benefit.

## Introduction

Gliomas account for 78% of primary malignant brain tumors (1). They originate from glial progenitor cells, namely astrocytes or oligodendrocytes, that constitute a significant portion of the mammalian brain. As such, gliomas are highly heterogeneous, known for their diverse histopathology, molecular genetics, and clinical behavior. In the United States, the incidence of gliomas varies from 4.7 to 5.7 per 100,000 persons (2), representing more than 18,500 new cases and 13,000 deaths annually (3). Glioblastoma multiforme (GBM), the most common and aggressive form of glioma, has a median survival of 16 months (4), carrying a five-year post-diagnosis survival rate of 6.8% (5). While the pathogenesis differs considerably, low-grade glioma has a more favorable timespan, ranging from 5.6 to 13.3 years, depending on several prognostic factors (6). Nonetheless, 70% of these tumors transform to GBM within 10 years (7). This eventually causes disability and premature death (8, 9).

Currently, the primary care pathway for gliomas is surgical resection followed by chemoradiotherapy, with concomitant temozolomide or other alkylating drugs (10, 11). Maximizing the extent of resection (EOR), until functional borders are encountered, is central to prolonging survival, improving the efficacy of adjuvant therapies, and delaying anaplastic transformation in both low- and high-grade glioma (12–15). The rationale for performing this type of “supratotal” resection is based on evidence that gliomas infiltrate the parenchyma well beyond magnetic resonance imaging (MRI)-defined abnormalities (16). Tumor recurrence may thus arise from undetected glioma cells growing beyond signal abnormalities, typically found 1–2 cm outside of contrast enhancement, as detected on volumetric fluid-attenuated inversion recovery (FLAIR) images. However, supratotal resection is not always practical or feasible to achieve.

Diffusely infiltrating gliomas often limit radical resection strategies, which preferentially invade along myelinated fibers in white matter tracts (17); cluster in eloquent brain regions with dense functional connections, like the basal ganglia and internal capsule (18); and develop functional multi-cellular network structures (19). Therefore, surgically-acquired lesions in functionally critical areas may cause significant neurologic morbidity and mortality (20, 21). Neural plasticity is another barrier to radical resection, due to functional reorganization (22, 23). Injury to white matter tracts, dynamically interacting with gliomas, is linked to post-operative deficits, accordingly (24, 25). Hence, the true benefit of resection depends on the “onco-functional balance”: (26) maximizing the extent of tumor removal while preserving patients’ functional integrity and quality of life.

Augmented reality (AR) is a technology that superimposes computer-generated, three-dimensional (3D) holograms, as well as auditory and sensory feedback, on reality in real time and space. This composite view of virtual objects with the real world creates a semi-immersive environment. Dating back to 1986 (27), AR has been applied in neurosurgery for nearly 30 years, carrying several advantages over conventional approaches (28).

First, AR maps patient-specific neuroanatomy directly onto the operating field, rendering display of surface and sub-surface targets. This has proven useful in visualizing anatomical structures, vasculature and hemodynamics, and deep-seated lesions in stereotactic, neurovascular, and tumor surgery, respectively (29). It also allows surgeons to access and contextualize radiological images and pre-operative planning. Second, AR eliminates attentional shifts between patients on operating tables and screens displaying relevant clinical information. This can reduce fatigue, cognitive load, and inattention blindness among surgeons, leading to more focused and efficient procedures (30). Third and finally, AR may disclose and compensate for intra-operative brain shift (31, 32): a highly prevalent and complex phenomenon of brain deformation due to changes in gravity and hydrostatic pressure, loss of cerebrospinal fluid, tissue manipulation or removal, and other factors (33). For image- and function-guided neurosurgery, this can invalidate patient-to-image registration and reduce the accuracy of localizing and resecting intra-cranial targets, as well as positioning surgical tools (34, 35). Thus, AR can be used to update virtual scenes, when combined with multimodal imaging and functional testing, to precisely identify pathologies, probe subcortical pathways, and tailor resection plans (28, 36, 37).

To date, applications of AR in neurosurgery have been limited to early clinical research. Given this stage, a variety of AR devices have been used for image projection, including head-up displays (HUDs), head-mounted displays (HMDs), microscopes, endoscopes, smartphones, and tablets (29, 37–40). Commercial display devices have also emerged, such as Google Glass (Google LLC, Mountain View, California, USA), HoloLens (Microsoft Corp., Redmond, Washington, USA), and Magic Leap (Magic Leap Inc., Plantation, Florida, USA) (29). Such innovation underscores the growing clinical and commercial interest in AR for neurosurgical practice, with proposed roles in skin incision, craniotomy, and resection (37, 38, 40). This is of particular import for eloquent brain tumors, as serious threats to human life and health, whereby AR may positively influence maximal safe resection and functional outcomes. In this review, we summarize current applications of AR in glioma surgery, as described in the scientific literature, with the aim of characterizing emerging trends and providing avenues for future research.

## Methods

We performed an in-depth systematic review, adhering to the Preferred Reporting Items for Systematic Reviews and Meta-Analyses (PRISMA) guideline (41). The review protocol was registered *a priori* with the Open Science Framework (OSF) (42), developed and maintained by the Center for Open Science (COS), which can be accessed via the digital object identifier (DOI): 10.17605/OSF.IO/DJ72P. The PRISMA 2020 Checklist (41), review strategy, and review protocol are additionally available for consultation upon reasonable request.

PubMed (National Library of Medicine), Embase (Elsevier), and Scopus (Elsevier) electronic databases were queried from inception to October 10, 2022, for relevant articles. The following search strategy was employed: PubMed: ((“augmented reality”[All Fields] OR “mixed reality”[All Fields]) AND (“glioma”[MeSH Terms] OR “glioma”[All Fields] OR “gliomas”[All Fields] OR “glioma s”[All Fields])); Embase: ((“augmented reality”/exp OR “augmented reality” OR “mixed reality”/exp OR “mixed reality”) AND (“glioma”/exp OR “glioma”) AND [article]/lim AND [humans]/lim); and Scopus: TITLE-ABS-KEY (((“augmented reality” OR “mixed reality”) AND (glioma))). No publication date or study type restrictions were applied.

Inclusion criteria consisted of (1) phantoms or patients of any age and biological sex diagnosed with glioma; (2) AR developed for or applied in glioma surgery, specifically to aid intra-operative navigation, localization, and/or resection; (3) protocol or technical note papers; case-control studies, case series, or case reports; retrospective, prospective, or concurrent cohort studies; or non-randomized, randomized, or post-hoc analyses of clinical trials; and (4) peer-reviewed studies published in the English language. In contrast, exclusion criteria comprised (1) phantoms or patients without glioma; (2) studies not developing or applying AR for/in glioma surgery, such as for patient education and surgical planning purposes; (3) reviews, editorials, expert opinion pieces, commentaries, letters to the editor, and articles with inaccessible full texts; and (4) studies not peer-reviewed and published in the English language. Duplicates were excluded prior to screening and studies that failed to meet full inclusion criteria were excluded from the overall analysis. The “Population, Intervention, Comparison, Outcomes, and Study design” (PICOS) (43) framework was applied for evaluating eligibility criteria in the qualitative synthesis.

Two independent reviewers, one with and one without prior content knowledge (N.B., M.H.Z.), screened articles against PICOS criteria, initially evaluating their titles and abstracts. Relevant studies were then selected for full text screening and assessed for eligibility. Inter-rater agreement was reported (Cohen's *k* = 0.74), with disagreements reconciled through discussion and/or by involvement of a third independent reviewer (A.R.) until a consensus was reached.

Another set of independent reviewers (W.H., A.K.U.) subsequently extracted data from eligible studies into a Microsoft Excel Spreadsheet (Microsoft Corp., Redmond, Washington, USA). Table cells were labeled as “Not applicable” (N/A) if data were missing. To ensure global data integrity, an independent reviewer (Z.O.U.) performed quality assurance checks at random. Data extracted from eligible studies included: study year, study location, study design, study type, number of total patients, number of glioma patients, number of phantom patients, glioma pathology, other pathologies, image acquisition phase, image data source, image segmentation technique, geometric modeling software, registration method, registration accuracy, display device, display brand, clinical application, primary outcomes, and any other pertinent findings. Following extraction, data were qualitatively described, using frequency (count, percentage), central tendency (mean, median, mode), and variability (range, standard deviation), as applicable, via R version 4.1.3 (44). Pooled statistical analyses, such as meta-regressions, were not performed due to heterogeneity in study designs and measured outcomes.

To assess the quality of evidence, a risk of bias assessment was conducted, using hierarchical classes of evidence in neurosurgery (45). This involved ranking the methodological rigor of each study, whereby “Level V” indicates the lowest or weakest level of evidence, such as case reports, and “Level I” indicates the highest or strongest level of evidence, such as randomized trials. As the study design becomes more rigorous, the quality of evidence increases, and the probability of bias decreases. Two independent reviewers (V.N.C., L.A.) conducted the risk of bias assessment, with inter-rater agreement reported (Cohen's *k* = 0.82).

## Results

The initial search query returned 77 articles for potential inclusion in the review [PubMed (*n* = 21), Embase (*n* = 25), and Scopus (*n* = 31)]. Of these, 40 (52%) unique articles remained following removal of duplicates (35, 46%) and inaccessible texts (2, 3%). After title and abstract screening, 15 (38%) articles were excluded for being literature reviews (7, 18%), investigating non-glioma tumors (1, 3%), not involving AR (4, 10%), or falling out of scope with PICOS criteria (3, 8%). Accordingly, 25 (63%) full text articles were assessed for eligibility, with 3 (12%) deemed non-eligible for inclusion.

**Figure 1:**
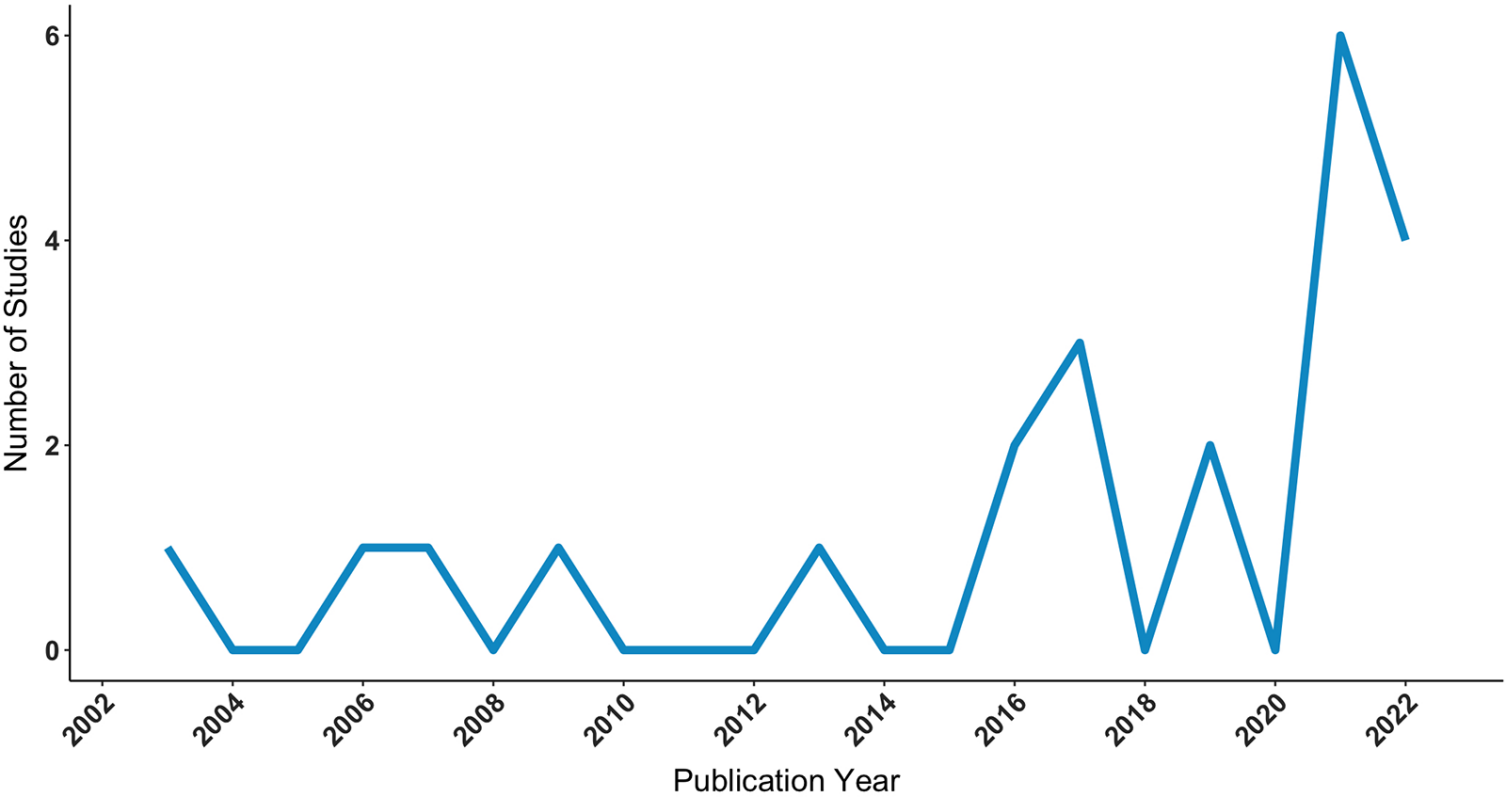
Number of studies applying AR in glioma surgery by publication year, as identified via PubMed, Embase, and Scopus electronic database searches, executed on October 22, 2022. AR, augmented reality.

**Figure 2:**
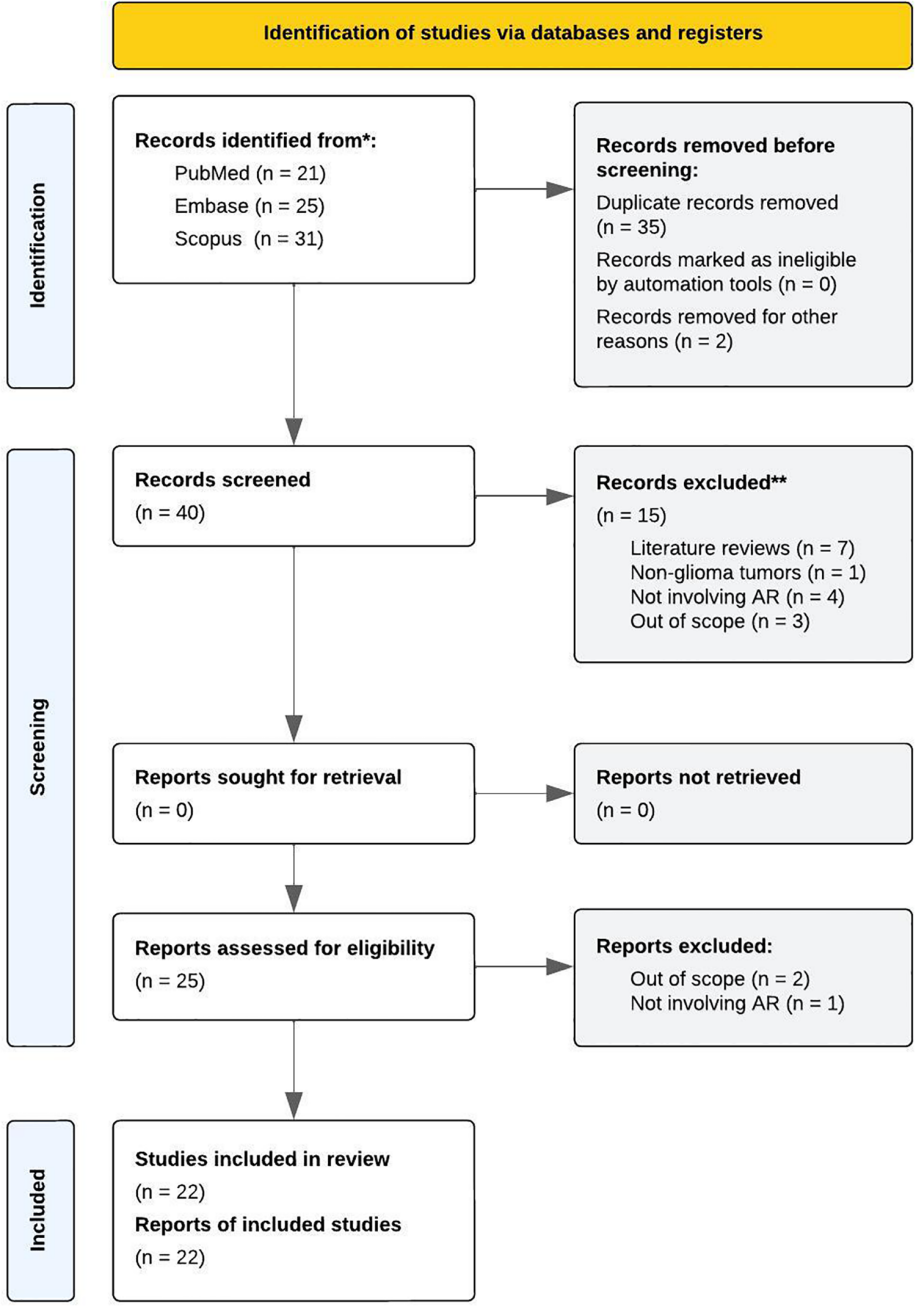
PRISMA 2020 flow diagram, describing the search strategy and selection schema of the review process.

The final review comprised 22 articles, with the first study published in 2003 (*n* = 1), the largest number published in 2021 (*n* = 6), and the most recent published in 2022 (*n* = 4). See studies by publication year in Figure 1. Regarding location, the majority of studies were conducted in Asia (13, 59%) followed by Europe (7, 32%) and North America (2, 9%). The PRISMA 2020 flow diagram, describing the search strategy and selection schema, is displayed in Figure 2. Characteristics of the studies included in the review are summarized in Tables 1–3.

**Table 1:** Characteristics of included studies.

Sample size	*n* (m)
Glioma patients	488 (22.2)
Phantom patients	22 (1.0)
Study type	*n* (%)
Development	10 (45.5)
Intervention	12 (54.5)
Imaging source	*n* (%)
3DRA	3 (13.6)
CT	16 (72.7)
CTA	3 (13.6)
DWI/DTI	10 (45.5)
fMRI	4 (18.2)
MRI	22 (100.0)
MR spectroscopy	2 (9.1)
Imaging phase	*n* (%)
Pre-operative	12 (54.5)
Intra-operative	2 (9.1)
Both stages	8 (36.4)
Modeling software	*n* (%)
3D Slicer	7 (31.8)
Amira	2 (9.1)
Avizo lite	3 (13.6)
BrainLab	5 (22.7)
Other	5 (22.7)
Not reported	1 (4.5)
Display Device	*n* (%)
Camera	5 (22.7)
Endoscope	1 (4.5)
HMD	3 (13.6)
HUD^a^	2 (9.1)
Microscope	7 (31.8)
Smartphone	2 (9.1)
Tablet	2 (9.1)
Video	1 (4.5)
Not reported	1 (4.5)
Study location	*n* (%)
China	5 (22.7)
England	1 (4.5)
Germany	3 (13.6)
Italy	2 (9.1)
Japan	8 (36.4)
Switzerland	1 (4.5)
United States	2 (9.1)
Levels of evidence^b^	*n* (%)
Level II	1 (4.5)
Level III	2 (9.1)
Level IV	13 (59.1)
Level V	6 (27.3)

3DRA, three-dimensional rotational angiography; CT, computerized tomography; CTA, computerized tomography angiography; DTI, diffusion tensor imaging, DWI, diffusion-weighted imaging; fMRI, functional magnetic resonance imaging; HMD, head-mounted display; HUD, head-up display; MRI, magnetic resonance; MRI, magnetic resonance imaging.

^a^ HUDs were used as integration tools for AR visualization in surgical microscopes.

^b^ Levels of evidence used by neurosurgeons in clinical practice.

**Table 2:** Design of included studies.

Author	Approach	Design^a^	Type	Sample	Glioma	Model^b^	Pathology
Archip et al. (46)	Prospective	Case Series	Development	*n* = 11	*n* = 11	*n* = 0	LGG, HGG
Carl et al. (47)	Prospective	Case Series	Intervention	*n* = 10	*n* = 2	*n* = 0	E, LGG
Chen et al. (48)	Prospective	Case Report	Development	*n* = 16	*n* = 1	*n* = 0	Glioma
De Mauro et al. (49)	Technical	Technical Note	Development	*n* = 0	*n* = 0	*n* = 0	LGG
Fick et al. (50)	Prospective	Case Report	Development	*n* = 3	*n* = 1	*n* = 0	GBM
Finger et al. (51)	Retrospective	Case Series	Intervention	*n* = 28	*n* = 6	*n* = 1	AA, DA, GG, PA
Ghimire et al. (52)	Retrospective	Case Series	Intervention	*n* = 180	*n* = 145	*n* = 0	LGG, HGG
Hou et al. (53)	Prospective	Case Series	Development	*n* = 35	*n* = 6	*n* = 0	DA, GBM, GG
Inoue et al. (54)	Prospective	Case Report	Development	*n* = 3	*n* = 1	*n* = 0	GBM
Iseki et al. (55)	Prospective	Case Series	Intervention	*n* = 148	*n* = 72	*n* = 0	Glioma
Koike et al. (56)	Prospective	Case Series	Intervention	*n* = 18	*n* = 18	*n* = 0	AA, AO, DA, GBM, OD
Koike et al. (57)	Prospective	Case Series	Development	*n* = 16	*n* = 16	*n* = 0	A, AOA, DA, GBM, OA, OD
Koike et al. (58)	Prospective	Case Series	Development	*n* = 15	*n* = 14	*n* = 0	Glioma
Liao et al. (59)	Prospective	Case Report	Development	*n* = 2	*n* = 1	*n* = 1	Glioma
Liu et al. (60)	Retrospective	Case-Control Study	Intervention	*n* = 53	*n* = 30	*n* = 0	A, GBM
Luzzi et al. (61)	Prospective	Case Report	Intervention	*n* = 1	*n* = 1	*n* = 0	GBM
Luzzi et al. (62)	Retrospective	Comparative Cohort Study	Intervention	*n* = 117	*n* = 54	*n* = 0	HGG
Mascitelli et al. (63)	Retrospective	Case Series	Intervention	*n* = 79	*n* = 4	*n* = 0	Glioma
Satoh et al. (64)	Prospective	Case Series	Intervention	*n* = 20	*n* = 7	*n* = 0	AE, DA, HGG, GG
Satoh et al. (65)	Prospective	Case Series	Intervention	*n* = 5	*n* = 3	*n* = 0	AA, GBM
Sun et al. (66)	Prospective	Comparative Study	Intervention	*n* = 134	*n* = 79	*n* = 0	LGG, HGG
Zhou et al. (67)	Prospective	Case Series	Development	*n* = 16	*n* = 16	*n* = 20	HGG

A, astrocytoma; AA, anaplastic astrocytoma; AE, anaplastic ependymoma; AO, anaplastic oligodendroglioma; AOA, anaplastic oligoastrocytoma; DA, diffuse astrocytoma; E, ependymoma; GBM, glioblastoma; GG, ganglioglioma; HGG, high-grade glioma (III, IV); LGG, low-grade glioma (I, II); OA, oligoastrocytoma; OD, oligodendroglioma; PA, pilocytic astrocytoma.

^a^ Study design reflects glioma patients only.

^b^ Models reflect phantom glioma patients.

**Table 3:** Summary of AR workflows in glioma surgery.

Author	Display	Brand	Technique	Registration	Accuracy	Primary outcomes
Archip et al. (46)	Surgical instruments	Not specified	Pre-operative MRI, fMRI, DTI, and/or MR spectroscopy data plus intra-operative MRI data segmented and modeled via 3D Slicer software, integrated into surgical instruments, superimposed on patient's brain	Patient-specific, volumetric non-rigid registration with anatomical landmarks and estimation for brain deformation	RD = 1.82 mm	Feasible application of non-rigid method that compensates for brain deformation within surgical time constraints; significantly increased alignment accuracy compared to rigid method; visualization of critical structural and functional brain areas
Carl et al. (47)	Microscope	PENTERO and PENTERO 900 (Carl Zeiss Meditec Inc., Oberkochen, Germany)	Pre-operative CT, CTA, and/or MRI data plus intra-operative CT data segmented and modeled via BrainLab software, integrated by HUD into surgical microscope, superimposed on patient's brain	Automatic, user-independent rigid and/or non-linear registration based on low-dose intra-operative CT via reference arrays and markers	TRE = 0.72 ± 0.24 mm	Reliable application with high accuracy; smooth integration into surgical workflow; good hand-eye coordination; intuitive depth perception and visualization of tumor extent and surrounding structures; high impression on patient anatomy, facilitating orientation
Chen et al. (48)	Smartphone	Honor 6 Plus (Huawei Technologies Co., Ltd., Shenzhen, China)	Pre-operative CT and MRI data segmented and modeled via 3D Slicer software, integrated into Android smartphone running Sina app superimposed on patient's head	Manual registration with anatomical landmarks and fiducial markers	D = 4.4 ± 1.1 mm	Practical application for visualizing and localizing supratentorial lesions, but not for infratentorial lesions; satisfactory accuracy compared to standard neuronavigation system; simple, cost-effective approach
De Mauro et al. (49)	Microscope	OPMI MD-NC1 (Carl Zeiss Meditec Inc., Oberkochen, Germany) (cited from prior work 68)	Pre-operative CT and MRI data segmented and modeled via 3D Slicer software, integrated into surgical microscope connected to infrared optical tracker of novel mixed reality system, superimposed on patient's head and brain	Manual, point-based registration with markers and reference model (cited prior work 68)	Not reported	Working prototype for visual and haptic simulation of LGG palpation; force feedback to distinguish normal from pathological tissue (VR); stereoscopic visualization with real time brain navigation and space cognition (AR)
Fick et al. (50)	HMD	Hololens 1.0 (Microsoft Corp., Redmond, Washington, USA)	Pre-operative CT and MRI data segmented and modeled via 3D Slicer software, integrated into HMD of holographic neuronavigation system, superimposed on patient's head and brain	Point-based matching rigid registration with anatomical landmarks, reference array, and visual markers	FRE = 8.55 mm	Proof-of-concept application for intraoperative patient tracking; relatively inaccurate registration and navigation accuracy, with hologram instability and drifting as well as functional difficulties
Finger et al. (51)	Endoscope	MINOP (Aesculap Inc., Tuttlingen, Germany)	Pre-operative CT or MRI data segmented and modeled via Scopus Nova Plan software, integrated into surgical endoscope of neuronavigation system, superimposed on phantom's or patient's head and brain (intraventricular space)	Hybrid registration with anatomical landmarks, surface matching, and optical reference matrix	D = 1.2 ± 0.4 mm	Feasible application with sufficient accuracy; increased precision to optimally place burr holes and apply trajectories; safe navigation and intuitive visualization of trajectories while perforating cortical and subcortical structures; helpful estimation of tumor location and surrounding structures
Ghimire et al. (52)	Microscope	KINEVO 900 (Carl Zeiss Meditec Inc., Jena, Germany)	Pre-operative MRI, fMRI, and/or DTI data segmented and modeled via StealthViz Medtronic software, combined with intra-operative ultrasound and cortical mapping data, integrated into surgical microscope of neuronavigation system, superimposed on patient's brain	Point-based registration with anatomical landmarks and visual markers	Not reported	Successful application; supplementary motor homunculus and novel subcortical motor map with accurate intra-operative identification of cortical and sub-cortical boundaries as well as localization of intercostal muscles
Hou et al. (53)	Smartphone	LVL CAM (Daniel LLC, Apple Inc., App Store)	Pre-operative CT and MRI data segmented and modeled via Windows XP software, integrated into iPhone smartphone running LVL CAM iOS app, superimposed on patient's head	CT- or MRI-based registration with markers; manual co-registration of virtual images with sagittal photograph based on anatomical landmarks via FUSED app	D ≤ 5 mm	Feasible application; useful for localizing intracranial lesions at low-cost with high accuracy; suitable for shallow supratentorial lesions of moderate size, but not for infratentorial lesions
Inoue et al. (54)	Camera	Qcam Pro 9,000 with headset [QCAM-200S-HS] and Qcam Connect (Logicool Co., Tokyo, Japan)	Pre-operative MRI and DTI data segmented and modeled via 3D Slicer software, integrated into handheld or headband web camera with optical markers connected to neuronavigation system, superimposed on patient's head and brain	Point-based registration with fiducial markers and reference table	FRE = 1.8	Feasible application; useful for visualizing and navigating corticospinal tract without damage; effective for resecting surface tumors, but not for deep-seated tumors due to camera malperformance; difficulty accurately judging depth perception
Iseki et al. (55)	Camera	Not specified	Pre-operative CT and MRI data plus intra-operative MRI data segmented and modeled, integrated into high-definition couple charged device camera on liquid crystal monitor of information-guided navigation system with optical tracking and real-time update, superimposed on patient's brain	CT- and MRI-based registration with markers	ME = 0.8 mm	Successful application with excellent accuracy; significantly increased average resection rate and total removal rate of malignant gliomas using open MRI with disclosed brain deformation and shift; improved EOR when simultaneously using real-time update navigation
Koike et al. (56)	Camera	Not specified	Pre-operative CT, MRI, DTI, and 3DRA data segmented and modeled via Avizo Lite software, integrated into fusion 3DCG combined with intra-operative brain surface photograph of patient, as part of mixed reality registration system	Manual registration via paired anatomical landmark and thin-plate spline methods using fusion 3DCG as reference	TRE = 0.70 mm	Successful application integrated with 3DCG tractography model; excellent accuracy despite brain shift at time of intra-operative photograph
Koike et al. (57)	Camera	Not specified	Pre-operative CT, MRI, and 3DRA data segmented and modeled via Avizo Lite software, integrated into fusion 3DCG combined with intra-operative brain surface photograph of patient, as novel mixed reality registration method	Automatic, non-rigid registration via paired anatomical landmark, thin-plate spline, and NMI methods using fusion 3DCG as reference	TRE = 0.72 ± 0.04 mm	Working method with highly precise spatial alignment between real and virtual space with angle versatility; 3DCG useful for skin incision and craniotomy planning with ability to display functional information
Koike et al. (58)	Camera	Nikon D500 (Nikon Corp., Tokyo, Japan)	Pre-operative CT, MRI, and 3DRA data segmented and modeled via Avizo Lite software, integrated into novel mixed reality image-guided system, projecting intra-operative brain surface photograph of patient onto 3DCG	Automatic registration using the NMI method to align intra-operative brain surface photograph (target) and 3DCG (reference) textures	TRE = 1.19 ± 0.14 mm	Feasible and cost-effective application of mix reality projection mapping with accurate alignment and minimal equipment; efficiently observed pre- and intra-operative information in the same coordinate system
Liao et al. (59)	Video	Not specified	Intra-operative MRI data segmented and modeled via fuzzy connectedness and 3D Slicer software, integrated into integral videography of image overlay navigation system, superimposed on phantom's or patient's head and brain	Semi-automatic, point-based registration with donut and fiducial markers	TRE = 0.90 ± 0.21 mm	Feasible application of real-time, autostereoscopic image overlay for open MRI-guided glioma surgery with simplicity and satisfactory accuracy; potential to reduce procedure time
Liu et al. (60)	HMD	HoloLens (Microsoft Inc., Redmond, Washington, USA)	Pre-operative CT, CTA, and MRI data segmented and modeled, integrated by HMD into MR holographic imaging technology system, superimposed on patient's head and brain	CT-based registration with visual markers and viewpoint tracking	Not reported	Successful application with real-time display of resection degree; significantly higher complete resection accuracy and post-operative recovery rate, as well as significantly lower post-operative complications compared to ultrasound
Luzzi et al. (61)	Microscope	KINEVO 900 (Carl Zeiss Meditec Inc., Oberkochen, Germany)	Pre-operative MRI, DWI, and DTI data segmented and modeled via BrainLab software, integrated into surgical microscope of neuronavigation system, superimposed on patient's brain	Not reported	Not reported	Safe and effective application in maximizing EOR of postcentral gyrus glioblastoma; high technique versatility; improved patient motor function; no tumor recurrence at 9-months follow-up
Luzzi et al. (62)	Microscope	OPMI Neuro-NC4 or KINEVO 900 (Carl Zeiss Meditec Inc., Oberkochen, Germany)	Pre-operative CT, MRI, fMRI, DWI, and MR spectroscopy data segmented and modeled via BrainLab software, integrated into robotic surgical microscope of neuronavigation system, superimposed on patient's brain	CT-based optical tracking registration with anatomical landmarks and surface matching	Not reported	Safe and effective application with significantly higher EOR and PFS rates compared to control group; optimized patient functional outcomes; limited accuracy and reliability due to parallax error and crowding of fiber tracts
Mascitelli et al. (63)	Microscope	PENTERO 900 (Carl Zeiss Meditec Inc., Dublin, California, USA) or Leica OH6 (Leica Microsystems Inc., Buffalo Grove, Illinois, USA)	Pre-operative CT, CTA, and MRI data segmented and modeled via BrainLab software, integrated by HUD (with variance) into surgical microscope, superimposed on patient's head and brain	Standard registration; co-registration of HUD with navigation system	Not reported	Safe application with good-to-excellent accuracy; useful for skin incision, craniotomy, dural opening, and corticectomy for intra-axial and superficial lesions; useful for bed/head positioning and extradural/intradural bone removal for skull base lesions; disabled in 59.6% of cases due to lack of use, distraction, and inaccuracy
Satoh et al. (64)	Tablet	Surface Pro (Microsoft Corp., Redmond, Washington, USA)	Pre-operative CT and MRI data segmented and modeled via Amira software, integrated into tablet PC with back-facing camera of trans-visible navigator system connected to optical markers, superimposed on patient's head and brain	Point-based registration with anatomical landmarks; co-registration of virtual and camera image	TRE = 2.31 ± 2.18 mm	Useful application for skin incisions, craniotomy, dural incisions, and superficial tumor resections; less useful for deep-seated tumor resections, except when using transcortical and interhemispheric approaches; minimal time and labor; pre-surgical discussions essential to efficacy
Satoh et al. (65)	Tablet	Surface Pro (Microsoft Corp., Redmond, Washington, USA)	Pre-operative CT and MRI data segmented and modeled via Amira software, integrated into tablet PC with back-facing camera of trans-visible navigator system combined with stereotactic frame, superimposed on patient's head and brain	Point-based registration with anatomical landmarks	Not reported	Feasible application to stereotactic biopsy of deep-seated lesions; clear trajectory and ability to advance biopsy probe precisely; avoidance of critical structures, the target point's location, and viewpoint turning
Sun et al. (66)	Microscope	Not specified	Pre-operative fMRI and DTI data plus intra-operative MRI and DTI data segmented and modeled via BrainLab software, integrated into surgical microscope of functional neuronavigation system, superimposed on patient's head and brain	Not reported	Not reported	Successful application for pre-operative planning as well as intra-operative guidance and manipulation; verified brain shift and quality control during surgery; significantly improved tumor resection rate and neurofunctional preservation
Zhou et al. (67)	HMD	HoloLens (Microsoft Inc., Redmond, Washington, USA)	Pre-operative CT, MRI, and DTI data segmented and modeled, integrated by HMD into novel stereotactic mixed reality-guided surgical navigation system, superimposed on phantom's or patient's head and brain	Markerless spatial registration with spatial drift and movement compensation methods to precisely align virtual anatomy with real patient pre-operatively	RMSE = 1.18 mm (phantom), 1.86 mm (patient)	Feasible application with suitable accuracy and efficacy for clinical use and resection; intuitive diagnosis and performance of surgical planning pre-operatively as well as identification of lesion boundary intra-operatively

3D, three-dimensional; 3DCG, three-dimensional computer graphics; 3DRA, three-dimensional rotational angiography; CT, computerized tomography; CTA, computerized tomography angiography; D, deviation; DTI, diffusion tensor imaging, DWI, diffusion weighted imaging; EOR, extent of resection; fMRI, functional magnetic resonance imaging; FRE, fiducial registration error; GBM, glioblastoma; ME, mean error; HGG, high-grade glioma; HMD, head mounted display; HUD, head up display; LGG, low-grade glioma; MR, magnetic resonance; MRI, magnetic resonance imaging; NMI, normalized mutual information; nTMS, navigated transcranial magnetic stimulation; PC, personal computer; PFS, progression-free survival; RD, residual displacement, RMSE, root-mean-squared error; TRE, target registration error.

**Figure 3:**
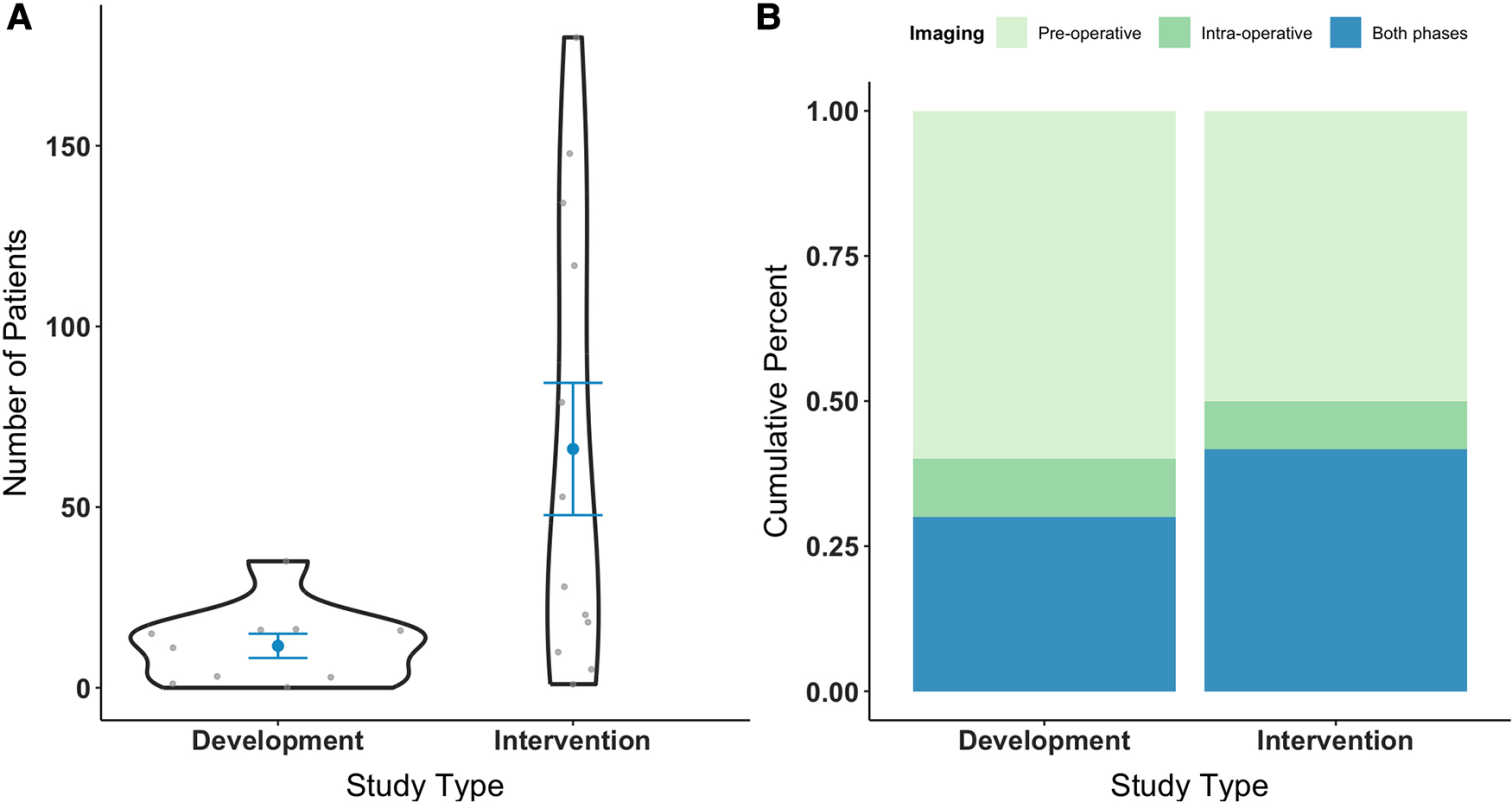
Number of patients diagnosed with glioma recruited in AR studies by study type, excluding phantom patients (**A**). Medical image acquisition phase by study type, spanning pre-operative and/or intra-operative stages (**B**). Study types are grouped by primary aim into “development” or “intervention”, with respect to AR application. AR, augmented reality.

At the time of data abstraction, based on primary aims, studies were sub-grouped into the following categories: “*development”* and “*intervention”*. Ten (45%) studies (46, 48–50, 53, 54, 57–59, 67) evaluated the technical design and suitability of AR for glioma surgery, principally assessing feasibility, accuracy, and/or reliability benchmarks (i.e., development). The remaining 12 (55%) studies (47, 51, 52, 55, 56, 60–66) investigated the clinical utility of AR for glioma surgery, with a focus on feasibility, safety, and/or efficacy profiles (i.e., intervention). Across studies, there was a total of 909 patients (41.3 ± 54.1), of which 488 were diagnosed with gliomas (22.2 ± 35.7). Other pathologies included meningioma, lymphoma, angioma, papilloma, craniopharyngioma, hemangioblastoma, and arachnoid cysts, among others. Figure 3A–B illustrates the number of glioma patients and imaging acquisition protocol across study types. Three studies (51, 59, 67) additionally leveraged phantoms (*n* = 22, 1.0 ± 4.3). Steps for applying AR models in glioma surgery comprised: (1) image acquisition, (2) image segmentation, (3) geometric model generation, (4) registration and tracking, and (5) intra-operative navigation via fused image overlay.

1. Image acquisition

Data for AR were obtained from a variety of medical imaging sources that occasionally integrated with network analysis and brain mapping. All studies performed anatomical or volumetric magnetic resonance imaging (MRI: 22, 100%), followed by computerized tomography (CT: 16, 73%), diffusion weighted or tensor imaging (DWI/DTI: 13, 59%), functional magnetic resonance imaging (fMRI: 4, 20%), computerized tomography angiography (CTA: 3, 14%), 3D rotational angiography (3DRA: 3, 14%), and magnetic resonance spectroscopy (MR spectroscopy: 2, 9%). Across image acquisitions, pertinent factors included spatial resolution, slice thickness, signal- and contrast-to-noise ratios, and image artifact.

**Figure 4:**
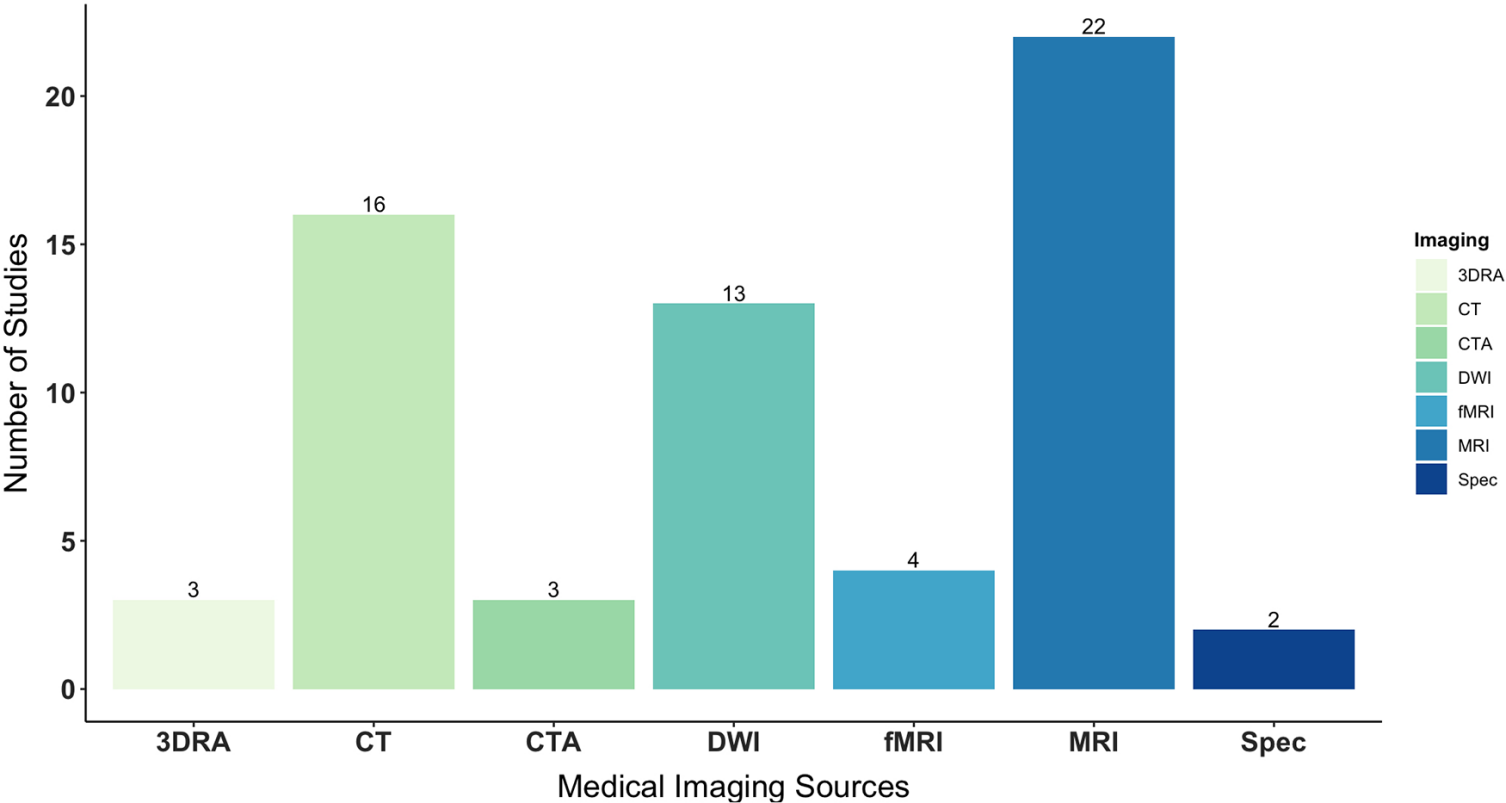
Types of medical imaging used as data sources for AR, specifically segmenting, modeling, formatting, and projecting virtual objects onto phantoms or patients’ real anatomy. AR, augmented reality.

DTI-based tractography, ultrasound, and navigated transcranial magnetic stimulation (nTMS) were additionally carried out as complimentary techniques in 8 (36%), 1 (5%), and 1 (5%) study, respectively. Of those involving DTI-based tractography, two studies (61, 62) performed high-definition fiber tractography with sodium fluorescein (HDFT-F), motivated by the prospect of increasing tumor resectability as well as survival rates. In another study (52), ultrasound images were obtained prior to corticectomy and following resection, with the aim of delineating lesion borders and post-resection cavity. See Figure 4 for the distribution of imaging sources used for AR. Most images were obtained pre-operatively (12, 55%), with 2 (9%) studies obtaining images intra-operatively and 8 (36%) from both phases (Figure 3B).

2. Image segmentation

Imaging data was partitioned, or “segmented”, into anatomical regions of interest, removing unnecessary and irrelevant information, commonly exported as Digital Imaging and Communications in Medicine (DICOM) images. This included target and adjacent structures, namely tumors and surrounding blood vessels, nerves, and other tissues; in addition to cortical and sub-cortical areas, such as the postcentral gyrus and corticospinal tract. In general, segmentation techniques involved thresholding, edge pixel detection, and region growing. Liao et al. (59) specifically developed a rapid, autostereoscopic segmentation method, using fuzzy connectedness for open MRI-guided glioma surgery.

3. Model generation

Three-dimensional modeling was used to convert segmentations to virtual objects, most frequently with 3D Slicer (7, 32%) and BrainLab (5, 23%) visualization software, based on DICOM images. To achieve this, tumor and cortical surface meshes, for instance, were exported as files suitable for 3D printing and computer-aided design (CAD), generally in stereolithrography (STL) file format. Unique to studies, Ghimire et al. (52) transformed positive motor responses, acquired from pre-operative nTMS, in 3D objects projected onto a tractography model of the corticospinal tract.

4. Registration and tracking

Registration was performed to format, align, and superimpose virtual objects—including drilling axis and cutting planes—onto patients' real anatomy. For studies using neuronavigation, this typically involved registering the patient to the system and co-registering the display device (e.g., surgical microscope) to determine the necessary transformation. In manual registration, virtual objects were scaled, translated, and/or rotated in relation to patients' head or brain, by the user, based on anatomical landmarks and fiducial markers. While this was the simplest approach, it was also the most time intensive and susceptible to human error, requiring continuous interaction between surgeons and technicians to update the AR scene. In automatic registration, landmarks and fiducials were often used as starting points with further processing and no user interaction. This approach was more expeditious, though relied on the quality of machine learning methods (i.e., training datasets). Registration also involved rigid, non-rigid, or hybrid surface interactions. Here, an assumption was made whether virtual and real objects—to be aligned—had the same shape, relating them by a single or multiple rigid transformations.

Given the variety and inconsistency in registration techniques, fused image overlay greatly varied across studies. Multiple terms were also used to express positional accuracy, or the estimate of error, as an indication of the system's ability to guide surgical targets. These terms included the target registration error (TRE), fiducial registration error (FRE), root-mean-squared error (RMSE), and target deviation (D). The TRE and FRE were the most widely calculated. Other factors contributing to variation comprised: geometric and optical distortions, such as incorrect tracking and display abnormalities; “swimming” effects, like bed movement and brain shift; and glioma presentation. Mascitelli et al. (63), for example, reported greater accuracy for superficial lesions compared to deep-seated ones (88.0% vs. 64.4%, *p* = .029). They also disabled HUDs in 59.6% of cases due to lack of use, distraction, and inaccuracy. Considering the aforementioned issues, several studies developed novel registration methods.

Fick et al. (50) designed a custom reference array, as an adjunct to HMDs, to correct initial registration for bed movements in GBM. While their technical workflow functioned as desired, and improved spatial understanding for surgeons, their registration accuracy was sub-optimal for clinical use (FRE = 8.55 mm). Relatedly, Archip et al. (46) evaluated a volumetric, non-rigid registration scheme to compensate for intra-operative brain shift. In 11 patients with eloquent supratentorial glioma, they revealed significant improvement in alignment accuracy compared to rigid-based, state-of-the-art technology (*p* < 0.001), with a mean residual displacement of D = 1.82 mm. Another study (67) leveraged a markerless spatial drift registration method to precisely align real and virtual objects. Pre-operatively, this aided surgeons in diagnosis and surgical planning; whereas intra-operatively, it helped them distinguish lesion boundaries and localize nerves, thereby increasing accurate resection of gliomas (RMSE = 1.86 mm). Liao et al. (59) similarly developed a spatial image registration method for integral videography image overlay, demonstrating satisfactory accuracy (TRE = 0.90 ± 0.21 mm). Nonetheless, there was high heterogeneity in registration methods and measurements, with no standard criteria for defining nor evaluating accuracy. Others failed to measure and/or report accuracy altogether (49, 52, 61–63, 65, 66).

5. Fused image overlay

**Figure 5:**
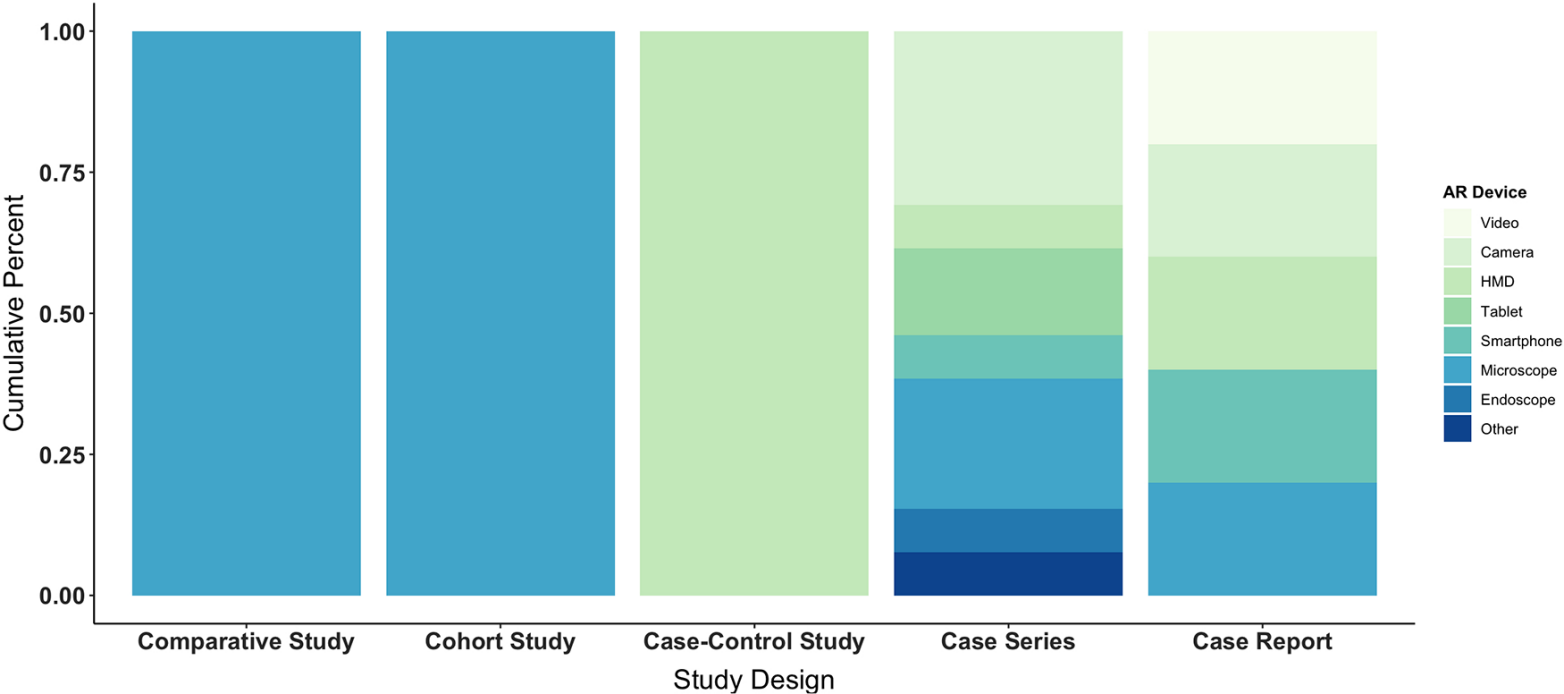
Types of augmented reality display devices used across study designs, including comparative, cohort, and case-control studies as well as case series and case reports. AR, augmented reality; HMD, head-mounted display.

Devices used to display virtual objects on the skull or dura of phantoms and patients included surgical microscopes (7, 32%), cameras (5, 23%), HMDs (3, 14%), tablets (2, 9%), smartphones (2, 9%), videos (1, 5%), and endoscopes (1, 5%). Of the studies utilizing surgical microscopes, two integrated AR via HUD (47, 63). Display device was unspecified for one study (46), which broadly referenced surgical instruments. See display devices by study design in Figure 5.

### Augmented reality applications

Iseki et al. (55) were among the first to evaluate AR in tumor surgery. An analysis of 42 patients with malignant gliomas, located in or adjacent to functional regions, showed markedly increased EOR (≥90%) when open MRI was simultaneously applied with real-time update navigation, which continuously refreshed intra-operative images. Using a similar concomitant method, Sun et al. (66) achieved complete resection in 69.6% of glioma patients (*n* = 79) compared to 36.4% of control patients (*n* = 55), with an average EOR of 95.2% ± 8.5% and 84.9% ± 15.7%, respectively (*p* < 0.01). The rates of post-operative recovery in motor, visual, and language function were also higher in the study group at two weeks and three months (*p* < 0.05). In both studies, intra-operative MRI disclosed and corrected for brain shift, providing surgeons with accurate and objective information as well as quality control during procedures.

A retrospective study (63) also showed potential for AR in intra-cranial surgery, detailing early experience with HUDs. For superficial and intra-axial lesions, HUD provided greater utility for skin incision, craniotomy, dural opening, and corticectomy, whereas the device was most useful for patient positioning and bone removal in those with skull base lesions. Although their sample with low-grade gliomas was small (*n* = 4), the authors postulated that HUD would be practical for guiding localization and resection, though more robust data was needed.

**Figure 6:**
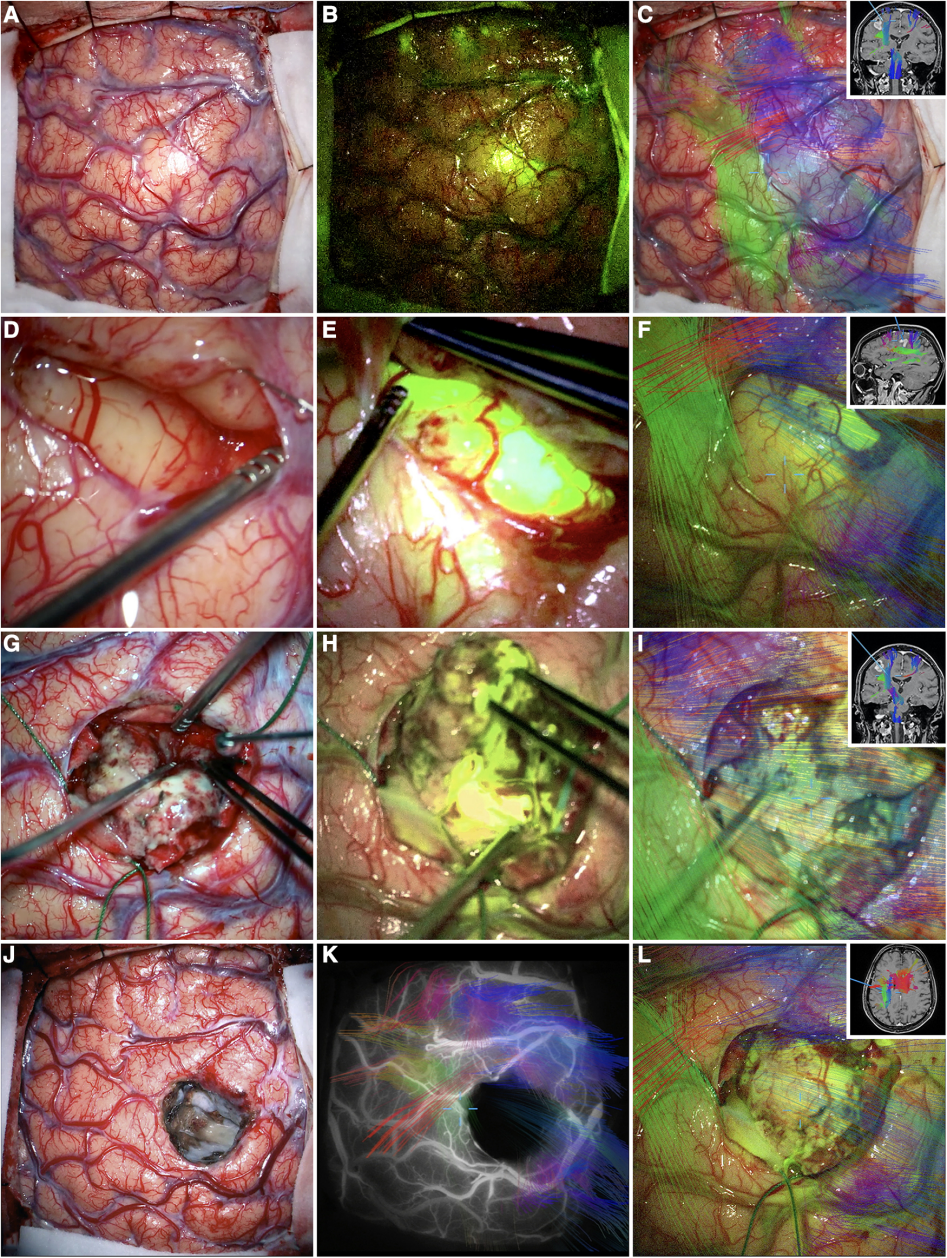
Illustrative case. Primary motor cortex glioblastoma of the dominant hemisphere. Intraoperative photographs obtained under white light (**A**), YELLOW 560 filter (**B**), and AR HDFT (**C**) before tumor resection. (**D–I**) The main steps of the surgery that were performed in large part along with the AR HDFT-F technique. (**J–L**) The surgical field at the end of the tumor resection obtained under white light (**J**), combined INFRARED 800 and AR HDFT during indocyanine green videoangiography (**K**), and AR HDFT-F (**L**). Insets in panels (**C,F,I,L**) are the screenshots obtained during the microscope focus-based neuronavigation. “Supratentorial High-Grade Gliomas: Maximal Safe Anatomical Resection Guided by Augmented Reality High-Definition Fiber Tractography and Fluorescein.” This figure is protected by Copyright, is owned by The Journal of Neurosurgery Publishing Group (JNSPG), and is used with permission only within this document. Permission to use it otherwise must be secured from JNSPG. Full text of the article containing the original figure is available at thejns.org.

Recently, Luzzie et al. (62) tested the safety and efficacy of a new multimodal AR technique. They compared patients with supratentorial high-grade glioma undergoing AR HDFT-F-based cytoreductive surgery (*n* = 54) to a cohort of patients undergoing conventional white-light surgery assisted by infrared neuronavigation (*n* = 63). See Figure 6 for an illustrative case. Results showed higher EOR (*p *= 0.019), lower post-operative neurological deficits (*p* = 0.011), and longer progression-free survival (*p* = 0.006) in the study vs. control group. The EOR was specifically ≥98% in 85% of study cases. However, the types, grades, and percentages of complications in both groups were analogous (9.2% vs. 9.5%).

This AR HDFT-F technique was further validated in the maximal safe resection of a postcentral gyrus GBM (61). Post-operatively, the patient reported significant improvement in upper extremity motor function and regained their ability to walk, with no recurrence at nine months follow-up. In a similar case, Inoue et al. (54) applied a newly developed AR neuronavigation system that superimposed tumors and vasculature plus motor tractography. This proved useful in visualizing the patient's lesion border and corticospinal tract, yet it had limitations in depth perception and accuracy. Their tumor resided at the corpus callosum inside the resection cavity as a result. However, no new neurological deficits were observed.

Two studies leveraged a tablet-based AR navigation system, called the “trans-visible navigator” (TVN). Satoh et al. (65) first applied this apparatus to stereotactic biopsy in three cases of deep-seated lesions, allowing surgeons to confirm target point accuracy and trajectory suitability, as well as precisely advance biopsy probes. This evaded the drawbacks of frame-based stereotactic navigation, resulting in satisfactory histopathology without complication. In a separate study (64), the TVN was applied in seven surgeries of low- and high-grade glioma, to which surgeons rated its utility across the neurosurgical workflow. Based on their findings, the apparatus was most practical for resecting superficial tumors, but less so for deep-seated ones, except when using transcortical and interhemispheric approaches. Results emphasized the importance of pre-operative discussions with surgeons in maximizing the effectiveness of AR.

Given the expense of modern navigation systems, two studies examined mobile AR (mAR) for localizing low- and high-grade glioma, as alternative low-cost solutions. Hou et al. (53) compared an iPhone-based method to a frameless neuronavigation system (*n* = 6), demonstrating technical feasibility with comparable accuracy (D ≤ 5 mm). Further, their device simplified image pre-processing, co-registration, and projection, all of which were completed under 10 min. Chen et al. (48) also examined mAR in the localization of a supratentorial glioma, leveraging the Sina Intraoperative Neurosurgical Assist app. Despite registration meriting improvement, their system was practical and reliable over standard neuronavigation (D = 4.4 ± 1.1 mm). Notably, both studies used manual registration in their AR workflows, likely increasing time and attenuating projection alignment, which the authors noted.

Other applications included intra-ventricular neuroendoscopy and intra-dural spinal surgery. Finger et al. (51) described their first experience with AR-guided neuroendoscopy among six patients with glioma in addition to one phantom model. By integrating pre-operative information into the endoscope's field of view, they were able to optimally place burr holes, estimate tumor location and surrounding structures, and apply trajectories for surgical intervention. Carl et al. (47), on the other hand, applied microscope-based AR via HUD in two patients with intradural spinal gliomas. In both study cases, AR provided intuitive visualization of tumor extent and neighboring structures, with high registration accuracy (TRE = 0.72 ± 0.24). This was particularly useful for visualizing the cranio-caudal extent of an intra-medullary ependymoma.

### Mixed reality applications

Six studies applied mixed reality (MR), a blend of augmented reality (AR) and virtual reality (VR), wherein physical and virtual objects co-exist and interact in real time. Motivated by computer-aided surgery (CAS), De Mauro et al. (49) developed a prototypical MR system for pre-operative training (VR) and intra-operative use (AR) embedded in a surgical microscope. Their VR feature simulated visual and tactile sensations of brain palpation, with force feedback interaction of soft and hard tissues. Using real patient data, this allowed surgeons to distinguish between normal and pathological tissue affected by low-grade glioma. In contrast, their AR feature enabled stereoscopic visualization of relevant 3D data for real-time brain navigation. This was specifically designed to aid image-guided neurosurgery.

Zhou et al. (67) similarly evaluated a novel MR navigation system. Using a markerless spatial registration method, they tested its ability to diagnose and perform surgical planning pre-operatively, as well as identify lesion boundaries intra-operatively. Compared to standard applications, and under ideal conditions, their system met accuracy, efficacy, and reliability benchmarks along with time requirements. This was validated in both phantom experiments (*n* = 20; RMSE = 1.18 mm; *M*_time_ = 6.02 min) and clinical trials (*n* = 16; RMSE = 1.86 mm; *M*_time_ = 7.95 min) among patients with high-grade glioma, indicating suitability for clinical use.

**Figure 7:**
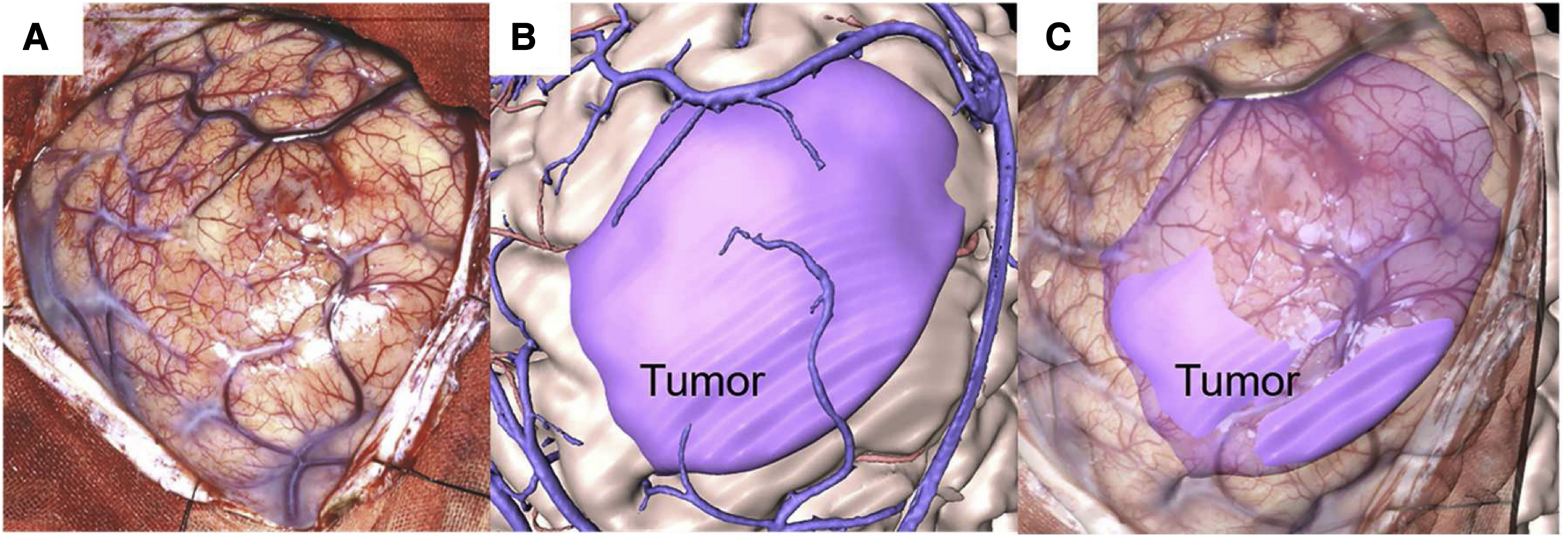
Illustrative case. A 31-year-old man with oligodendroglioma. (**A**) Intraoperative brain surface photograph in JPEG (Joint Photographic Experts Group) format. (**B**) Fusion 3-dimensional computer graphics (3DCG) created from preoperative imaging studies. The purple highlight indicates the tumor area. (**C**) Mixed-reality computer graphics created by aligning the intraoperative brain surface photograph and fusion 3DCG. The purple highlight indicates the tumor area. “Development of Innovative Neurosurgical Operation Support Method Using Mixed-Reality Computer Graphics.” © 2021 The Author(s). Published by Elsevier Inc. Licensed under CC-BY-NC-ND.

Moreover, using MR projection mapping (MRPM), Koike et al. (58) developed an image-guided surgery system that projects intra-operative brain surface photographs (BSPs; real space) onto high-resolution 3D computer graphics (3DCGs; virtual space). See Figure 7. This was accomplished without the need for large-scale equipment, such as neuronavigation and other computer-assisted technologies. In 14 glioma patients, their system displayed accurate alignment of patient anatomy (BSPs) and medical images (3DCGs), presented in the same coordinate system, even after brain shift due to craniotomy (TRE = 1.19 ± 0.14 mm). Alignment accuracy was evaluated by two neurosurgeons, together under consultation, who measured the difference between BSPs and 3DCGs. This was performed after dividing craniotomy areas into 16 fields. Further, MRPM made it possible for surgeons to plan trajectories for intervention based on cortical stimulation mapping. No post-operative complications were observed.

In a follow-up study (*N* = 15) (51, 52), recruiting patients with left hemispheric glioma, MRPM allowed surgeons to visualize the spatial correlation between medical images and the surgical field, specifically language-function hubs in the frontal lobe (TRE = 0.70 mm). This was likewise achieved despite brain shift at the time of the intra-operative BSP. Both studies leveraged a registration method previously developed and validated by Koike et al. (57), which demonstrated high spatial accuracy (TRE = 0.72 ± 0.04 mm).

In a retrospective, case-control study, Liu et al. (60) compared MR holographic imaging technology (*n* = 30) to ultrasound (*n* = 23) in neurosurgery for spinal cord glioma. Findings showed a significantly higher total tumor resection rate in the experimental group than in the control group (96.7%, vs. 82.6%; *p* < 0.05). This extended to more accurate complete resections (93.3%, vs. 73.5%; *p* < 0.05), far lower post-operative complications (3.3%, vs. 21.7%; *p* < 0.05), and improved recovery rates at 12 months follow-up (56.7%, vs. 41.1%; *p* < 0.05). Additionally, the tumor recurrence rate was lower in the experimental group compared to the control group at 12 months follow-up (0.0%, vs. 4.3%); however, this was not significant (*p* > 0.05). Enhanced MRI results were used to master the accuracy of intra-operative complete resections, informed by prior literature on AR and anatomic pathology (69). Post-operative MRI was then used to evaluate complete resections and their rate, as well the incidence of complications after surgery. Functional recovery was assessed via Modified McCormick Scale (MMS) grading.

## Discussion

In this review, we summarized applications of AR in glioma surgery. Qualitatively, AR is a valuable tool that precisely overlays multiple imaging datasets, plus other relevant clinical information, onto the surgical field in real time. This obviates the need for surgeons to shift focus away from patients to nearby monitors for guidance, and mentally relate 2D information into 3D anatomy. Inattention blindness and interpretation error can therefore be mitigated. Further, AR enhances visualization of tumor complexity and its relationship to critical structures. This facilitates spatial understanding of neuroanatomy; aids surgeons in navigating, localizing, and resecting lesions; and may subsequently lead to improved patient outcomes.

However, at present, there is limited data that AR effectively extends the EOR and PFS as well as preserves motor, visual, and language function post-operatively. This is evidenced by the number, breadth, and quality of published studies. Of the 22 included in this review, 10 studies developed and tested AR systems, including segmentation and registration techniques, while the remaining 12 applied AR interventionally. Notably, 86% of studies were of low-level or weak evidence, based on hierarchical classes of evidence in neurosurgery (45), largely comprising case series and case reports. Accordingly, 77% of studies included 18 glioma patients or less and 73% lacked standard control groups. No studies involved random sampling nor random assignment.

Another critical limitation observed here, relevant to future research, was the inability to “double blind” patients and surgeons a key method for reducing detection bias. This problem underlies any technical advancement in surgery, namely trials of non-pharmacological treatments with physical components (70–73). As a result, surgeons who trusted in the efficacy of AR may have—unconsciously or deliberately—influenced the EOR. This might have led to an overestimation of treatment effects and perhaps more significant outcomes. Blinding of outcome assessors was additionally absent and/or unreported; yet it serves an important role in the case of soft endpoints, such as psychosocial function and quality of life. Still, this is not readily achieved (70, 72, 74). A possible solution is to apply the IDEAL Framework (75, 76), a paradigm for incorporating evidence-based advances in neurosurgery. Here, specific study designs and reporting standards are recommended across five stages of surgical innovation: Idea, Development, Exploration, Assessment, and Long-term study. A controlled, interrupted-time series design is one acceptable alternative, suggested by the IDEAL framework, for minimizing known bias (77).

From a technical standpoint, AR workflows considerably varied across studies. Diverse approaches were employed to acquire and segment images, model virtual objects, register and track systems, and display fused data intra-operatively. This variation extended to measurements, with several terms used to express registration error, or the accuracy of overlaid virtual objects on patients’ head and/or brain, each with its own shortcomings. The TRE, for instance, measures the anatomical region of interest for surgeons in 3D space, yet has poor depth perception. Other indices fail to correlate with (TRE) or underestimate (TE) true accuracy. This lack of standardization in measurement and reporting is well-documented in the field (78), and likely affects the validity of AR and its ability to guide localization and resection of intra-cranial targets, as well as the positioning of surgical instruments. Registration thus presents a significant and pressing challenge to precision neurosurgery. Along the same accord, most studies relied on software and hardware not formally vetted for pre- and/or intra-operative use. This chiefly applied to novel AR systems and techniques, which may lead to publication bias. As such, reported outcomes, at this stage, must be interpreted with caution.

One strategy towards standardization is founding a consortium of AR workflows in neurosurgery, including image acquisitions and open-source software, that could be utilized across research institutions. Compiling larger, more homogeneous, and longitudinal datasets may help identify universal methods; and allow for the development, validation, and use of machine learning algorithms (79)—and complimentary techniques (e.g., ultrasound 80, 81)—to maximize accuracy throughout procedures. This space also stands to benefit from a “readiness framework” to evaluate AR suitability for surgical implementation, drawing parallels to Tang et al.'s (82) analytic model. Their team specifically developed an evidence-based schema for assessing AR in medical education, underscoring four criteria: quality, application content, outcome, and feasibility. Adapting this model to AR in glioma surgery may address inconsistency in assessment tools and reliably gauge clinical utility. Mastering the use of AR in educational, training, and pre-operative settings will likewise increase its intra-operative value.

## Limitations

This systematic review carries inherent limitations. First, no automated tools were employed during the selection and data collection process, increasing susceptibility to human error. In the event this occurred, studies returned by databases may have been overlooked, incorrectly excluded, and/or misreported in this review. Second, ascertaining the quality of evidence for each study was subjective, despite following hierarchical classes for neurosurgery. Studies may have been misclassified accordingly, especially those meeting criteria for more than one level. However, additional quality assurance measures, outlined in our methodology, were taken to ensure global data integrity. Finally, our qualitative analysis is limited by its data, which is heterogeneous at best. Standards for measurements and reporting will help improve the therapeutic value of AR in glioma surgery moving forward, and will enable meta-analytic approaches to precisely estimate both technical performance and treatment effects.

## Conclusion

AR has increasing potential in the surgical management of glioma. It enables improved understanding of complex relationships between anatomy and pathology, aiding in real-time intra-operative navigation, localization, and resection. Further, there are signals of improvement in neurofunctional preservation associated with AR use, pointing to real, discernable benefit to patient care. This is evermore salient given the poor prognosis of gliomas, especially those with malignant and invasive presentations. However, technical and design limitations are readily apparent. A universal approach for developing, applying, and measuring AR systems, for instance, is critically absent. The field must consider the importance of consistency and replicability to effectively converge on standard uses of AR and its therapeutic value.

## Data Availability

The original contributions presented in the study are included in the article/Supplementary Material, further inquiries can be directed to the corresponding author.
